# Artificial Intelligence Implementation in Healthcare: A Theory-Based Scoping Review of Barriers and Facilitators

**DOI:** 10.3390/ijerph192316359

**Published:** 2022-12-06

**Authors:** Taridzo Chomutare, Miguel Tejedor, Therese Olsen Svenning, Luis Marco-Ruiz, Maryam Tayefi, Karianne Lind, Fred Godtliebsen, Anne Moen, Leila Ismail, Alexandra Makhlysheva, Phuong Dinh Ngo

**Affiliations:** 1 Norwegian Centre for E-Health Research, 9019 Tromsø, Norway; 2 Department of Mathematics and Statistics, Faculty of Science and Technology, UiT The Arctic University of Norway, 9037 Tromsø, Norway; 3 Institute for Health and Society, Faculty of Medicine, University of Oslo, 0318 Oslo, Norway; 4 Department of Computer Science and Software Engineering, College of Information Technology, United Arab Emirates University, Al Ain 15551, United Arab Emirates; 5 National Water and Energy Center, United Arab Emirates University, Al Ain 15551, United Arab Emirates; 6 School of Computing and Information Systems, Faculty of Engineering and Information Technology, The University of Melbourne, Parkville, VIC 3010, Australia

**Keywords:** artificial intelligence, machine learning, CFIR, AI implementation, eHealth, healthcare, deep learning, diagnosis, prognosis

## Abstract

There is a large proliferation of complex data-driven artificial intelligence (AI) applications in many aspects of our daily lives, but their implementation in healthcare is still limited. This scoping review takes a theoretical approach to examine the barriers and facilitators based on empirical data from existing implementations. We searched the major databases of relevant scientific publications for articles related to AI in clinical settings, published between 2015 and 2021. Based on the theoretical constructs of the Consolidated Framework for Implementation Research (CFIR), we used a deductive, followed by an inductive, approach to extract facilitators and barriers. After screening 2784 studies, 19 studies were included in this review. Most of the cited facilitators were related to engagement with and management of the implementation process, while the most cited barriers dealt with the intervention’s generalizability and interoperability with existing systems, as well as the inner settings’ data quality and availability. We noted per-study imbalances related to the reporting of the theoretic domains. Our findings suggest a greater need for implementation science expertise in AI implementation projects, to improve both the implementation process and the quality of scientific reporting.

## 1. Introduction

Nowadays, artificial intelligence (AI) has become ubiquitous, and much more advanced and user-friendly than it was two decades ago. In many respects, AI has become reliable and permeates many aspects of our daily lives, such as face and speech recognition apps. Yet, only recently have we seen a corresponding rate of adoption in the healthcare services. AI systems can emerge as a smart solution to reduce clinical staff workload in a world with increasingly saturated healthcare systems. AI is different from simple technology interventions in the sense that AI does not just manage data, but it provides suggestions and recommendations directly shaping the clinical decision process [1,2].

There exists a wide body of literature on the barriers to and facilitators of implementing AI in healthcare [3,4,5]. However, much of what we know about these barriers and facilitators comes from anecdotal evidence [6], narrative commentaries [7] and reviews [8,9,10,11], mostly without any empirical support or sound theoretical basis. As a result, the determinants of AI implementation success in healthcare are still poorly understood [12]. We lack a complete overview of all the factors that are relevant to implementing AI in clinical settings. In this study, we turn to implementation science [13] to analyze the facilitators and barriers, based on accounts from existing implementations.

Implementation science is a fairly new field, whose emerging theories, models and frameworks have the potential to inform our understanding of AI implementation in a more widely accessible and systematic way. This multidisciplinary approach, combining AI and implementation science, transcends the traditional boundaries of each of the fields. Blending these two disparate, yet complementary, fields is key to our understanding of AI implementation in healthcare. However, there is a need to reconcile the methodological differences and conflicting domain-specific jargon. In the next two subsections, we explore the fundamental aspects of each of these two fields.

### 1.1. Artificial Intelligence

AI is not a new concept, but renewed interest in the field is widely attributed to the increasing abundance of digital data and the advancements in data analytic approaches. AI comprises many different areas that range from logic-based models to machine learning (ML). Logic-based models [14] have been successfully used in areas such as biomedical ontologies management (e.g., SNOMED-CT automatic concept classification [15]) and decision support (e.g., SAGUE, Arden syntax, GLIF, etc. [16]). Conversely, ML has had a less prominent role, partially due to the lack of health data availability for training data-driven algorithms. Data-driven methods have the capacity to unveil patterns in data that otherwise would remain hidden [17]. They stand a comparatively better chance at dealing with subpopulations, where one clinical guideline may not suffice to provide the optimal treatment (e.g., multimorbid patients).

In the scope of this study, we refer to AI as systems that are used to solve healthcare problems of interest and are powered by ML. Witten et al. [18] define ML as ”a family of statistical and mathematical modeling techniques that use a variety of approaches to automatically learn and improve the prediction of a target state, without explicit programming”. This definition precludes most expert systems and other basic knowledge-based AI systems that use simple rule-based processes or Boolean rules.

### 1.2. Implementation Science

In a seminal paper, Eccles and Mittman [13] define implementation science as “…the scientific study of methods to promote the systematic uptake of research findings and other evidence-based practice into routine practice…”. In contrast, AI, comprised mostly of computing sciences, defines implementation as generally referring to development of software components according to a specification, for example, implementing an algorithm. To the extent that these two fields define implementation in significantly different ways, their focus as academic fields will also diverge markedly. Computing sciences focus more on developing artefacts rather than systematically studying how the artefacts are put into routine use. This lack of shared meaning will inevitably have serious consequences for search strategies to find relevant articles in academic databases. For the purpose of discussion in this study, we use the definition of implementation from implementation science.

### 1.3. Pilot Study vs. Implementation Trial

In the context of screening for implementation trials, there is a thin line between a pilot study and an implementation trial. Pilot studies and feasibility studies are necessary components in the path to implementation. Curran et al. [19] describe a progressive path from efficacy studies, followed by effectiveness studies and then proceeding to implementation research. Pearson et al. [20] distinguish between studies conducted for testing effectiveness and studies intended to evaluate implementation strategies, using three conceptualizations named Hybrid Type 1, Type 2 and Type 3. These conceptualizations are based on Curran et al.’s [19] work on combining both effectiveness studies and implementation science elements. Major distinctions are made between the purpose of the study and the methods used. The primary purpose of Hybrid Type 1 is for testing the clinical or public health effectiveness of an intervention. Hybrid Type 2 considers both the clinical effectiveness and evaluation of an implementation strategy. The primary goal of Hybrid Type 3 is to evaluate the effectiveness of the implementation strategies, with a secondary goal to observe other data such as health outcomes. In the current study, we focus on studies evaluating implementation strategies (Hybrid Type 2 and 3), where a full implementation already exists or where the organization is committed to a full roll-out, and the smaller implementation trial forms part of a risk minimization strategy. Thus, pilot studies that fall under Hybrid Type 1 are excluded.

### 1.4. Objectives

The goal of this scoping review is to characterize the barriers and facilitators influencing the implementation of ML methods in the healthcare setting. This study differs from the existing reviews in at least two major ways. First, whereas the existing reports on barriers and facilitators are fragmented, this study analyzes these barriers and facilitators in a more systematic and theoretic way, which allows us to identify reporting problems and knowledge gaps. Second, the existing reviews do not discriminate based on the phases of implementation. Therefore, most of the studies include algorithm development, efficacy and effectiveness studies. The current study, on the other hand, focuses on empirical observations from the late phases of implementation and roll-out.

## 2. Methodology

This scoping review follows standard reporting, based on the Preferred Reporting Items for Systematic Reviews and Meta-Analyses (PRISMA) extension for scoping reviews [21] (see Additional file S4—PRISMA checklist). A scoping review is an appropriate methodology for exploring new areas of research [22]. There are only a few implementations, and reviewing auxiliary information sources, such as reports or websites of the implementation, adds value to our overall understanding of the implementation context. The authors are a multidisciplinary team of statisticians, data scientists, computer scientists and clinicians. The authors have had experience in the implementation of data-driven ML methods and their performance evaluation.

### 2.1. Protocol

Since this review is scoping in nature and required an additional search phase, no protocol was published in advance. However, the data extraction form was designed before starting the search.

### 2.2. Eligibility Criteria

The screening goal was to exclude articles that do not study actual or real-world implementations. To identify and understand the barriers and facilitators, based on empirical observations and real experiences with production systems, we included implementation trials and excluded early pilot testing of algorithms. Table 1 shows the eligibility criteria based on the population, intervention, comparator, outcomes and study type (PICOS). We searched for papers published between 2015 and 2021. Only publications in English were included.

### 2.3. Information Sources and Selecting Sources of Evidence

The databases and indexes that we searched included PubMed, IEEE, ACM, Google Scholar, and the Web of Science. These sources represent the major indices of scientific articles related to both AI–ML and the healthcare sciences.

In addition to scientific publication databases, we used other sources of information, such as the database of FDA-approved AI systems [23]. We performed an additional Google search on the Internet to gain a better understanding of both the functions of the system and the implementation context. We used website information and any available reports relevant to the specific implementation. These auxiliary information sources are appropriate for use in a scoping review and helped us screen the studies. However, the data extraction was only based on scientific articles.

### 2.4. Search Query and Two-Phase Search

As a multidisciplinary team of researchers, we knew about the conflicting definitions of implementation. However, we could not anticipate the extent of the problem or how it would affect our search results. We defined an iterative search process with two phases. In the first phase, we searched only the title with terms such as “implement*” and “practice”. Through a limited screening of the title and abstract, we quickly realized that many potentially relevant papers were missed, and most of the papers were about implementing algorithms. In phase two of the search, we had to define our search more broadly; include both title and abstract, and more synonyms. This iterative approach to a search strategy is supported by the literature [24].

We identified a broad spectrum of studies, and, given the lack of a unified vocabulary for indexing relevant articles, we had to make a subjective judgement regarding where a study fell on a continuum: (i) algorithm implementation, (ii) efficacy, effectiveness or algorithm validation, (iii) implementation trial, or (iv) full implementation. An overwhelming majority of the search hits fell within (i) and (ii). Only the studies identified as class (iii) or (iv) were included in this study.

The basic structure of the search query was «Artificial intelligence AND implementation AND healthcare». Synonyms and terms related to AI were then added using the logical disjunction operator (OR). The initial abstract screening was done using Rayyan [25]. All the search strings are available in [Additional File S1—search string]. An example of the search in PubMed was as follows:

(«machine learning»[Title/Abstract] OR machine learning[mesh] OR «artificial intelligence»[Title/Abstract] OR artificial intelligence[mesh] OR «deep learning»[Title/Abstract] OR deep learning[mesh] OR «neural network»[Title/Abstract] OR «image analysis»[Title/Abstract] OR «deep neural networks»[Title/Abstract] OR «supervised learning»[Title/Abstract] OR «unsupervised learning»[Title/Abstract] OR «reinforcement learning»[Title/Abstract] OR «automated algorithms»[Title/Abstract] OR «adaptive algorithms» [Title/Abstract]) AND (implement* [Title] OR practice [Title] OR approved [Title]) AND (y_10[Filter]))

### 2.5. Data Extraction and Items

The data extraction variables were developed through weekly brainstorming sessions. At least four co-authors (TC, TOS, MT, PDN) participated in each brainstorming session, defining the list of topics relevant for extraction. The initial sessions were focused on the free definition of the topics and variables useful for extraction. As the brainstorming sessions advanced, the categories of variables were inductively defined, leading to the final list of agreed variables for data extraction as shown in Table 2. The final list of extraction items was calibrated through limited tests by four co-authors.

### 2.6. Critical Appraisal of Individual Sources of Evidence

We used the Mixed Methods Appraisal Tool (MMAT) [26] to critically assess the quality of the included studies. MMAT was an appropriate tool because the nature of relevant studies varied widely between qualitative, quantitative and mixed methods. Three co-authors (TC, MATH, LMR) assessed the quality of the studies and disagreements were resolved by discussion.

### 2.7. Synthesis of Results

**Figure 1:**
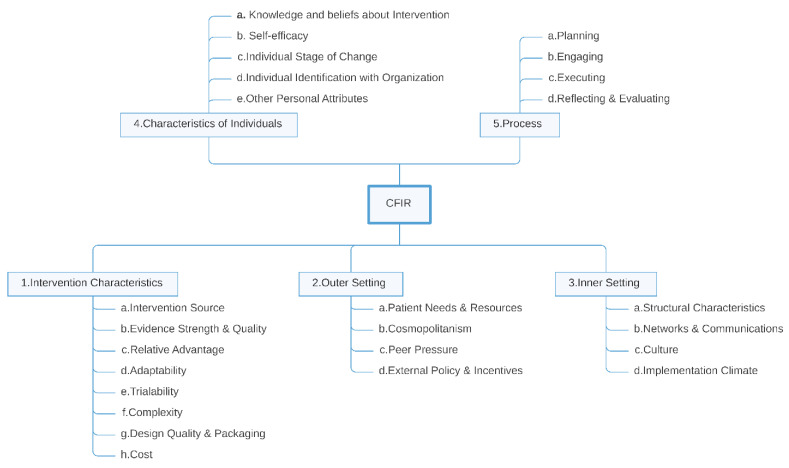
Theoretical constructs of the Consolidated Framework for Implementation Research (CFIR).

Qualitative methods were used to synthesize the extracted facilitators and barriers based on the Consolidated Framework for Implementation Research (CFIR) (see Figure 1 and the codebook in Additional file S5). CFIR is a framework used by many implementation research studies. It provides an index of constructs for organizing findings in a consistent and understandable manner [27]. It naturally invites us to follow a deductive strategy in the synthesis of results. However, due to the many technical and organizational details found in AI implementations, we considered that a more granular presentation was convenient in the synthesis of results, as previous studies in the field of clinical decision support (CDS) had shown [28]. To that end, we opted for a mixed approach, aiming to join the proven coherency of the CFIR constructs, for the general classification of barriers and facilitators, with the detailed approach that open inductive coding provided for defining items about the specific context under examination. In this way, we broke down the details of each CFIR construct in the framework’s codebook into more granular sub-constructs that were easily mappable to specific barriers and facilitators in AI implementations. With this rationale in mind, we split the analysis of results in two stages and performed a mixed inductive-deductive approach.

### 2.8. Open Inductive Coding and Mapping onto the CFIR Framework

Five co-authors (LMR, TOS, MATH, MT, PDN) read the full papers, extracting any section that pointed to a possible barrier or facilitator. Free comments (e.g., observations and interpretations) from the reviewers were allowed. All the papers were reviewed by at least two co-authors. Both text segments and free comments were imported into the qualitative analysis software, MaxQDA [29], for further analysis.

Two co-authors (TOS, LMR) went through the extracted segments of the selected papers independently. Initially, a deductive approach to code the segments into CFIR constructs was used. After one iteration, the constructs were considered not granular enough. Then, two reviewers (TOS, LMR) proceeded with an inductive approach, with no predefined code list. The reviewers marked all the segments of text that indicated a barrier or a facilitator for AI adoption.

Once all the papers had been coded, the reviewers met with three other members of the team, who had read all the papers but had not coded the texts. Iterative meetings were performed to go through all the coded texts and crosscheck the results. Equivalent labels were merged into one single concept when agreements were found. Any disagreements were discussed until all the members agreed on the optimal concept to code a specific fragment of the text by checking the full text and re-reading the section of interest. The usual sources of disagreement were the scope of one concept and the specific barriers and facilitators that one concept should encompass.

The same concept could be described as a barrier or as a facilitator by different studies (for instance, data quality was described as a barrier with ”insufficient data quality“ and a facilitator as ”availability of high-quality data“. This process resulted in an index of concepts that fully categorized all the barriers and facilitators found in the full texts. The index of concepts evolved iteratively until the end of this inductive analysis, refining the semantics of each concept and its scope.

The index of concepts was analyzed by the team and mapped into the constructs of the CFIR framework. Any disagreements about which CFIR construct was the most appropriate for the concept were resolved by discussing the possible options until an agreement was reached. With regards to coverage, the CFIR fully covered the concepts defined in our index, and all of the index concepts could be mapped to CFIR constructs.

## 3. Results

### 3.1. Selection of Sources of Evidence

**Figure 2:**
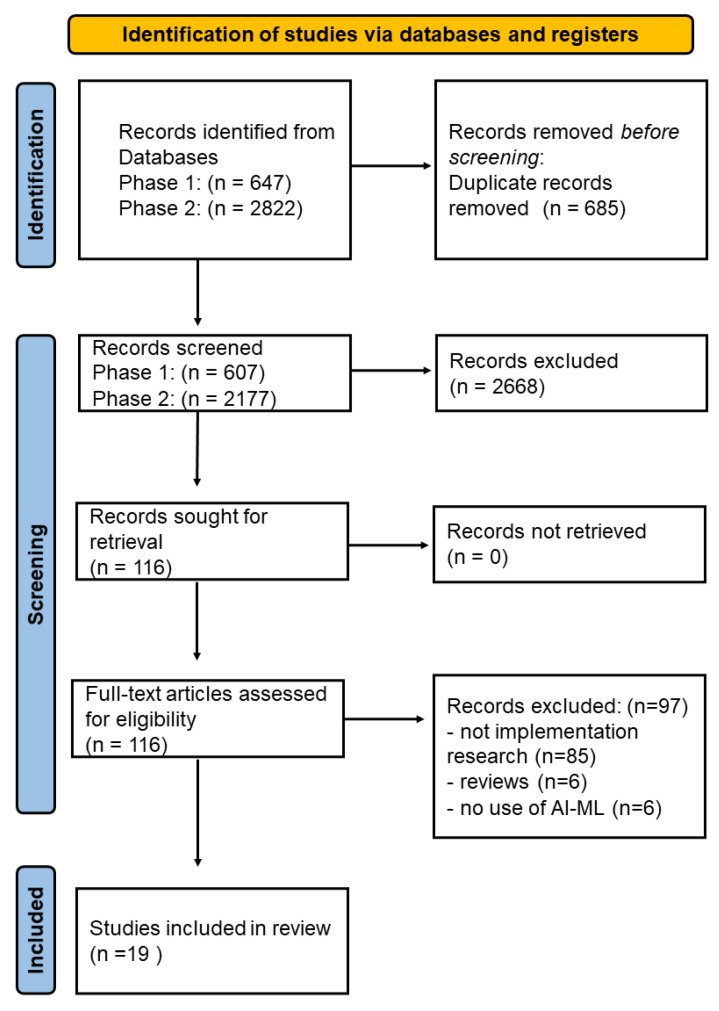
PRISMA flow diagram based on the template by Page et al. [30]. Study characteristics and critical appraisal.

Phase one of the search resulted in a total of 607 articles, while the second phase resulted in 2177 articles, after the removal of duplicates. Four co-authors (TC, TOS, MT, PDN) independently screened the titles and abstracts according to the inclusion and exclusion criteria. This resulted in the removal of 2668 articles, leaving 116 relevant articles. A full-text assessment was conducted on these 116 relevant articles, which resulted in 19 included articles, as shown in Figure 2, 11 of which were published in 2020.

As shown in Table 3, about half of the included studies were conducted in the USA and Canada (47%, *n =* 9/19); about a quarter (26%, *n =* 5/19) were conducted in Northeastern Asia, and only three in Europe. Several medical fields were represented: sepsis (16%, *n =* 3/19), diabetes (11%, *n =* 2/19), cardiology (11%, *n =* 2/19), mental health (11%, *n =* 2/19), emergency care (11%, *n =* 2/19) and palliative care (5%, *n =* 1/19), and the rest were for all patients (16%, *n =* 3/19). The most common medical task (an AI-use case) was screening (79%, *n =* 15/19). Only two of the studies had AI systems targeted towards use by patients, while the rest were meant to be used by clinicians or healthcare staff (89%, *n =* 17/19). In terms of AI algorithms, the majority of the studies applied deep learning (63%, *n =* 12/19).

In terms of the appraisal, three co-authors (TC, LMR, MATH) used the MMAT template to independently appraise the included studies (see Additional file S2—MMAT), and any disagreements were reconciled through discussion. Except for one study, all the other studies had well-defined research questions and sufficient data to address the questions they posed. We categorized the studies into quantitative non-randomized (58%, *n =* 11/19), qualitative (37%, *n =* 7/19) and quantitative descriptive (5%, *n =* 1/19). In all the quantitative studies, the participants were representative of the target population. For all but one of the qualitative studies, the methods used were appropriate to answer the posed questions.

### 3.2. Results of Individual Sources of Evidence

The concepts identified through the inductive process were further classified into ten broader themes: evaluation and testing, background, management and engagement, data quality and management, trust and transparency, clinical workflow, interoperability, finance and resources, technical design and AI policy and regulation. As illustrated in Table 4, most of the facilitators were based on the management and engagement theme (47%, *n =* 27/57). None of the reviewed articles reported barriers related to management and engagement. The second most common facilitators were related to the theme of evaluation and testing (14%, *n =* 8/57), while a third were related to technical design (12%, *n =* 7/57). For the barriers, the most common were interoperability issues (19%, *n =* 7/36), data quality and management (17%, *n =* 6/36) and trust and transparency (14%, *n =* 5/36).

Table 5 shows the concepts extracted from each study. Three studies had no easily discernible barriers or facilitators [34,36,41]. Three studies that reported facilitators did not report any barriers [38,39,47], and two studies that reported barriers did not report any facilitators [33,35].

### 3.3. Mapping Extracted Concepts to CFIR

**Figure 3:**
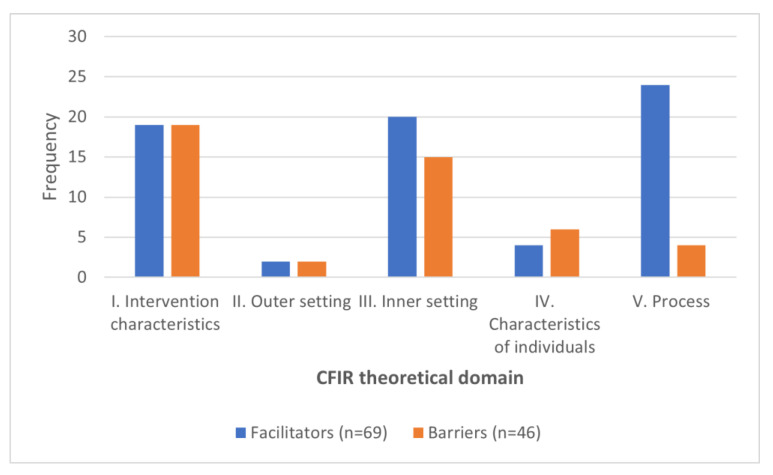
Frequency of facilitators versus barriers.

The mapping between the coded concepts and the corresponding CFIR constructs is available in [Additional file S3—CFIR mappings]. In total, 69 facilitators and 46 barriers were identified and coded following the CFIR framework. The result of this mapping is summarized in Figure 3. Most of the studies reported V. Process as the main facilitator for AI implementation in healthcare (35%, *n =* 24/69). The second most popular facilitators were based on III. Inner setting (29%, *n =* 20/69), followed by I. Intervention characteristics (27%, *n =* 19/69). Most of the barriers were reported for I. Intervention characteristics (41%, *n =* 19/46), followed by III. Inner setting (33%, *n =* 15/46) and II. Outer setting, which had the least number of both barriers and facilitators.

## 4. Discussion

Viewed in total, the reporting of facilitators and barriers related to the I. Intervention characteristics, III. Inner setting and V. Process appear somewhat balanced, with some under-reporting of the II. Outer setting and the IV. Characteristics of individuals. Viewed per study, however, the reporting imbalances are more apparent, and we highlight two kinds of imbalance. The first case relates to how a study can concentrate on a single theoretic domain and neglect the rest, and the second is where, regardless of the theoretic domain, a study focuses on either one of facilitators or barriers.

In the first case of theoretic domain imbalance, some studies focused on the characteristics of the intervention [33,49], while others focused on the process [40,47]. Consequently, we end up with an incomplete picture of the implementation for any single study, and it is difficult to compare findings across studies [50]. In the second case, which was typically the case, the studies focused more on the facilitating factors than the barriers. In extreme cases, a study might focus on facilitators alone [38,39,47] and completely neglect the barriers, or the other way round [33,35]. We attribute this poor reporting and imbalance to a lack of implementation science expertise.

We further describe some of the salient highlights in each of the five CFIR domains. These highlights are based on the frequency of discussion they generated in the included studies. The major facilitating factors were related to the implementation process itself and the involvement of users, and this finding is consistent with the literature [51]. In contrast, barriers were mostly associated with the intervention characteristics and inner setting, specifically interoperability, trust and transparency and non-availability of high-quality data.

### 4.1. Intervention Characteristics

#### 4.1.1. Evidence Strength and Quality

ML algorithms need to continuously learn from new data. As data change and as methodological techniques advance, so must the models, and this presents several challenges. One of challenges is the continuous need to validate algorithms and test whether their specificity and sensitivity have deteriorated. This partially explains why many of the included studies conducted fresh validation tests.

In the validation process, it is essential to make sure the training data represents the population to which the AI system is applied. In practice, results may not be representative across populations [43,48], and experiences may not be generalizable to a new setting [40]. Projects that have been properly evaluated and tested are more likely to succeed in the implementation process. In this regard, [49] recognized clinical trials and multi-center studies are a necessary part of implementation [33,49].

#### 4.1.2. Design Quality and Complexity

Technical design decisions might affect the implementation by facilitating or hindering this process. Usability was cited as both an important facilitator and a barrier. For instance, Romero-Brufau et al. [43] faced problems related to the documentation and presentation of results, and reported difficulties understanding patient information from the decision support system. They dedicated two months to refining the interface of the system and adapting it to the workflow, reflecting the importance of customization in the implementation process. Intuitive, unintrusive, and easy-to-use systems have better chances to succeed in the implementation process [42,45].

#### 4.1.3. Interoperability, Adaptability and Generalizability

In order to successfully implement an AI system in a clinical workflow, the system must interoperate with the targeted hospital systems. In [46], the lack of data interoperability was exposed when overworked nurses were asked to print and deliver medical histories in paper form. This resulted in a situation where medical histories were often not printed, and thus were not provided to physicians. Data interoperability issues led to poor integration in the clinical workflow.

#### 4.1.4. Integration with Clinical Workflow

A lack of integration with the clinical workflow can be a barrier to the implementation process. For example, Sendak et al. [40] reported workflow issues with model retraining and updating, which are intrinsic ML processes. In addition, projects that are too technically complex or disruptive are at risk of hindering the implementation process:

“Models that require additional work, even if it is as little as looking at another screen and clicking a few more times, are much less likely to be implemented or sustained”

[44].

As a solution, some studies showed that ML-based methods compatible with logic-based CDS methods are easier to integrate in the clinical workflow. An example is the use of neural networks for knowledge discovery during the development stage, where results have been later discretized as Arden syntax ECA rules in the production stage [35].

### 4.2. Outer Setting

#### External Policies and Incentives

The outer setting was discussed by only one study [35], mostly from the perspective of the legislative environment as a barrier, and the study was conducted in Europe, where AI algorithms used in healthcare are considered Software as a Medical Device (SaMD) and require CE-certification by law. This certification is expensive and time-consuming. However, an exemption allows AI software under clinical evaluation to be used without CE conformity. This requires only an approval from an ethical board and a study protocol adhered to for auditing. This exemption is generally utilized due to costs related to certification [35].

### 4.3. Inner Setting

#### Resource Availability

The availability of high-quality data resources within the organization was discussed as an important determinant factor. Most of the studies used electronic health records (EHR) as the primary source of data, and they reported their complexity and inadequate use as a barrier [37,42]. Due to the complex nature of the EHR, key data that can be used to predict the outcome of interest is not always available or ready in the structured format for AI algorithms [31,37,43].

“…key data that reliably predict the outcome of interest may not be readily available as structured, discrete data inputs from the EHR…”

[43]

Missing data, noisy data, or data without proper labels and identifiers were among the main factors that lowered data quality and were consequently reported as barriers. Besides data quality, the frequency of data updates is another important issue in maintaining the validity of predictive models [33]. Although data quality and management were usually seen as a barrier by most of the studies, Lee et al. [31] mentioned that rich data availability was a facilitator of the implementation process.

### 4.4. Characteristics of Individuals

#### Knowledge, Beliefs and Other Personal Attributes

This domain speaks to the perceptions and beliefs of the individuals involved in the implementation. For instance, a lack of trust among clinicians might hinder the implementation process. The clinicians must trust that the system maintains good sensitivity and specificity and provides trustworthy suggestions in line with evidence-based practice and clinical judgement. As Sendak et al. [40] note:

“Clinical leaders prioritized positive predictive value as a performance measure and were willing to trade-off model interpretability for performance gains”.

At the beginning of the implementation process in [46], the physicians showed interest in the use of an AI-based decision support system that improves diagnostics. However, two of them reported errors in the medical histories, which led them to a wrong diagnosis. As a consequence of sharing those reports among the physicians, the decision support system was perceived as prone to error, generating persistent distrust, and so undermining the usefulness of the system.

Another factor is explainability, a characteristic that directly conditions the transparency and trust of the AI implementation, which, in turn, are precursors of privacy and fairness [52]. No explainability technique is a one-size-fits-all solution for every intervention. Each AI system needs to adapt its explainability to the context and the audience using the model. For instance, a CDS based on a logistic regression model is perfectly understandable by clinicians, but it may be opaque in the context of a patient-oriented app. Other models, such as neural networks, are generally opaque and could be complemented with recent discoveries in explainability techniques such as feature relevance or visualization [53,54,55].

### 4.5. Process

#### Champions and Key Stakeholders

User involvement ranked as the most reported facilitator, followed by the education of key stakeholders. In the very beginning of a project, it is useful to have a common justification [47] and an early mapping of the workflow [43]. In order to attain this, it is necessary to get the relevant participants on board as early as possible [43,44,48]. The stakeholders’ feedback and involvement, especially from the leadership, clinicians and users, are also necessary throughout the implementation process [32,38,46,48]. In many instances, the projects strongly supported by the leadership have a higher probability to succeed. Senior leadership support can be crucial to achieve a shared vision among different stakeholders to reach the desired impact.

### 4.6. Implication of the Results and Recommendations for the Future

The barriers and facilitating factors emerging from this study are not surprising, since they are widely reported in the literature. The included studies presumably have overcome many of the barriers since the studies are based on the late stages of implementation. We expected that insight into the determinants of their successes would shed new light on our basic understanding of AI implementation in clinical settings. However, what we uncovered was insufficient and imbalanced reporting of some key theoretical domains, which suggests a lack of implementation science expertise in the reporting of relevant projects.

The traditional recommendation for e-health implementation processes is to involve both ICT and clinical domain experts. All the included studies seem to have followed this basic recommendation, but our findings suggest there still is a missing piece of the puzzle-socio-organizational considerations. Considering the successes of implementation science as a field, perhaps it is time we looked beyond these traditional recommendations in order to uncover additional synergies based on new modes of inquiry native to implementation science, integrating insights from social science theories and abstractions.

We also showed that domain differences between AI and implementation science have an impact on multidisciplinary research. Since implementation has become a key aspect of AI in healthcare, it is important to unify the vocabulary to make relevant research more accessible to both fields. This could start with annotating relevant publications with an appropriate keyword indicating the implementation stage or purpose of the study, for example, using Curran et al.’s [19] Hybrid Types or research pipeline model (ibid.). Classifying implementation stages is an important problem [56] and may reduce the ambiguity of terminology and bridge the gap between data science and implementation science.

### 4.7. Limitations

Perhaps one of the major limitations of this study is the uncertainty regarding coverage of the relevant literature, which was conditioned by multiple factors. First, we noted that some AI implementations might not have been subject to rigorous scientific study or evaluation, while other implementations were only reported locally in internal reports. This made the implementations essentially inaccessible. In addition, ambiguity related to terminology was a huge factor in successfully identifying all the relevant studies. We allude to the difficulties of defining implementation and the consequences it had on our search strategy and screening.

It is possible that attentional bias is a factor in our findings. Since we set out to identify advanced implementations, it is conceivable these implementations faced comparatively fewer challenging barriers than those of a typical implementation. This might partially explain why there were many more facilitating factors than barriers. In looking at successful implementations, it is quite possible we missed many important barriers from failed implementations.

## 5. Conclusions

This study exemplifies a theory-based approach to synthesizing determinants of AI implementation success and formalizes known gaps and biases related to how AI implementations are reported. In addition to highlighting the major facilitators and barriers, we noted a widespread imbalance and insufficient reporting of AI implementations in clinical settings. We single out the II. Outer setting and IV. Characteristics of individuals as two key theoretical domains, which were not fully explored in the included studies. As a result, we know very little about the knowledge and beliefs, self-efficacy and other personal attributes of the people involved in the implementations. Similarly, any policies, incentives, collaborative networks or competitive pressures that helped or hindered these implementations are largely unknown. These factors represent an important knowledge gap and require further inquiry before AI implementation in healthcare can be more fully understood.

Further, we recommend two remedial actions based on our findings: (i) implementation science expertise should be a part of every AI implementation project in healthcare in order to improve both the implementation process and the quality of scientific reporting, and (ii) scientific publications involving AI implementations in clinical settings should be annotated with an implementation stage or purpose to make relevant research more easily accessible.

## Figures and Tables

**Table 1:** Study eligibility criteria based on PICOS.

	Inclusion	Exclusion
Population	Humans Any disease groups	Animals
Intervention	Use ML techniques Implemented in healthcare settings or approved by the FDA, EMA or equivalent body	Systems without ML component Purely logic-based systems
Comparator	Not relevant	Not relevant
Outcomes	Implementation results or evaluation Implementation barriers Implementation facilitators Economic benefits, process improvement, treatment outcomes, etc.	Papers without implementation results (other than technical and algorithmic performance)
Study type	Prospective validation in clinical settings Retrospective implementation evaluation Published between 2015 and 2021	Review papers, expert opinions Non-English papers Pilot and experimental studies without real-world implementation

**Table 2:** Data extraction items.

Data Type	Examples
Study	authors, year, title, journal
Description	country of implementation, product name, company/research group, timeline for implementation, implementation phase
Role of AI	patient group, primary users, training required, medical specialty, medical task
Technology	AI methods, algorithms, hardware, transparency, interpretability, explainability
Data	type of input, sample size for training
Ethics	security and privacy, bias, other ethics issues
Clinical Validation	type, sample size
Legal	process for approval, approval status, other legal issues/processes
Barriers Facilitators	qualitative methods used to extract the barriers and facilitators

**Table 3:** Properties of the included studies.

Study (Year)	Country	Medical Field	Medical Tasks (Problem)	Primary Users	AI Techniques
Lee [31] (2015)	USA	Emergency Dept. patients	Screening	Clinicians, nurses, planners	Machine learning
McCoy [32] (2017)	USA	Sepsis	Screening	Clinicians and nurses	Machine learning
Moon [33] (2018)	Korea	Delirium	Screening	Clinicians	Logistic regression
van der Heijden [34] (2018)	Netherlands	Diabetes/retinopathy	Screening	Clinicians	Deep Learning
Schuh [35] (2018)	Austria	All patients	Screening	Clinicians	Deep learning, fuzzy logic, decision tree
Guo [36] (2019)	China	All patients	Screening	Patients	Deep learning
Cruz [37] (2019)	Spain	Cardiology, Gastrointerology, Psychiatry	Quality improvement	Clinicians (GPs, Pediatricians)	Deep learning
Joerin [38] (2019)	USA/Canada	Psychology	Treatment	Staff, patients and family caregivers	Natural language processing
Gonçalves [39] (2020)	Brazil	Sepsis	Screening	Nurses	Deep learning
Sendak [40] (2020)	USA	Sepsis	Screening	Clinicians	Deep Learning
Gonzalez-Briceno [41](2020)	Mexico	Diabetes/retinophathy	Screening	Clinicians	Deep Learning
Xu [42] (2020)	China	All patients	Screening	Nurses and clinicians	Deep learning
Cho2020 [20]	Korea	Cardiology	Screening	Nurses and clinicians	Deep learning
Romero-Brufau [43] (2020)	USA	All patients	Screening, prognosis, treatment	Clinicians, outpatient care coordinators	Decision tree
Scheinker [44] (2020)	USA	Chronic kidney disease, diabetes	Screening, prognosis, treatment	Clinicians	Deep learning
Davis [45] (2020)	USA	Radiology	Screening	Clinicians	Deep learning
Petitgand [46] (2020)	Canada	Emergency Dept.	Diagnose	Clinicians	Deep learning
Betriana [47] (2021)	Japan	Mental health	Treatment	Patients (receiver) nurse (controller)	Not specified
Murphree [48] (2021)	USA	Palliative care	Screening	Palliative care team (clinicians)	Gradient Boosting Machine (GBM)

**Table 4:** Inductive extraction of concepts and themes.

Theme	Facilitators	Barriers	Concept
Evaluation and testing	8	3	-
Background	5	2	Experiences and prior knowledge, Prior evidence, Healthcare demand
Management and engagement	27	-	External collaboration, Planning, Feedback incorporation, Communication, Involvement, Motivation, Leadership, Education of workforce, Patient needs, Champions
Data quality and management	1	6	Data availability, Data quality
Trust and transparency	1	5	Interpretability, Trust
Clinical workflow	4	4	Integration, Disruptiveness (alert fatigue)
Interoperability	2	7	Model Interoperability, Data interoperability, Generalizability
Finance and resources	1	3	Available Resources, Cost
Technical design	7	4	Usability, Documentation and presentation of results, Adaptability, Innovation, Complexity, Trialability
AI policy and regulation	1	2	Organizational policy and culture, Regulation and law
**Totals**	**57**	**36**	

**Table 5:** Facilitators and barriers based on the concepts.

Study	Facilitators	Barriers
Lee [31]	Healthcare demand, Evaluation and testing, Generalizability, Data availability, Available Resources, Trialability, Motivation	Regulation and law
Betriana [47]	Healthcare demand, Planning, Education of workforce, Involvement, Evaluation and testing	--
Cho [49]	Generalizability, Evaluation and testing	Evaluation and testing, Interpretability, Model interoperability
Cruz [37]	Evaluation and testing, Integration, Leadership, Usability	Data availability
Davis [45]	Integration, Usability	Evaluation and testing, Trust
Gonçalves [39]	Motivation, Experiences and prior knowledge	--
Joerin [38]	Involvement, Evaluation and testing, Patient needs, Adaptability	--
McCoy [32]	Healthcare demand, Communication, Feedback incorporation, Education of workforce	Disruptiveness (alert fatigue)
Moon [33]	--	Model Interoperability, Data quality
Murphree [48]	Involvement, Communication	Generalizability
Petitgand [46]	Involvement, Organizational policy and culture	Data interoperability, Usability, Documentation and presentation of results, Trust
Romero-Brufau [43]	Planning, Involvement, Education of workforce, Adaptability	Usability, Data quality, Data availability, Generalizability, Evaluation and testing
Scheinker [44]	Prior evidence, Involvement, Planning, Evaluation and testing	Trust, Complexity, Disruptiveness
Schuh [35]	--	Data quality, Experiences and prior knowledge, Cost, Regulation and law, Data interoperability
Sendak [40]	Involvement, Planning, External collaboration, Leadership, Integration, Interpretability, Evaluation and testing, Champions, Education of workforce	Cost, Trust, Available Resources, Generalizability, Prior evidence, Integration
Xu [42]	Education of workforce, Evaluation and testing, Innovation, Usability, Integration	Data availability, Integration
Gonzalez-Briceno [41]	--	--
Guo [36]	--	--
van der Heijden [34]	--	--

## Supplementary Materials

The following supporting information can be downloaded at: https://www.mdpi.com/article/10.3390/ijerph192316359/s1, Additional File S1—Search strings; Additional File S2—MMAT critical appraisal; Additional File S3—CFIR mappings; Additional File S4—PRISMA checklist; Additional File S5—CFIR codebook.
