# Supplementary material for: Artificial Intelligence Implementation in Healthcare: A Theory-Based Scoping Review of Barriers and Facilitators
Source: Int J Environ Res Public Health. 2022 Dec 6;19(23):16359. doi: 10.3390/ijerph192316359 (PMC9738234; doi:10.3390/ijerph192316359)
Supplement: Supplementary file 1 [file ijerph-19-16359-s001.zip › Additional file S3.pdf]

CFIR domain	CFIR construct	Concept	Theme
I. INTERVENTION CHARACTERISTICS	B. Evidence and strength and quality	Evaluation and testing	Evaluation and testing
		Prior Evidence	Background
	C. Relative advantage	Innovation	Technical design
	D. Adaptability	Model Interoperability	Interoperability
		Generalizability	Interoperability
		Adaptability	Technical design
	E. Trialability	Trialability	Technical design
	F. Complexity	Disruptiveness (alert fatigue)	Clinical workflow
		Complexity	Technical design
	G. Design quality and packaging	Data quality	Data quality and management
		Generalizability	Interoperability
		Usability	Technical design
		Documentation and presentation of results	Technical design
	H. Cost	Cost	Finance and resources
II. OUTER SETTING	A. Patient Needs and Resources	Patient needs	Management and engagement
	B. Cosmopolitanism	External collaboration	Management and engagement
	D. External Policies and Incentives	Regulation and law	AI policy and regulatory
III. INNER SETTING	B. Networks and communication	Communication	Management and engagement
	C. Culture	Organizational policy and culture	AI policy and regulatory
	D. 1. Tension for change	Healthcare demand	Background
	D. 2. Compatibility	Data quality	Data quality and management
		Integration	Clinical workflow
		Disruptiveness (alert fatigue)	Clinical workflow
		Data interoperability	Interoperability
	D. 4. Organizational Incentives and Rewards	Organizational policy and culture	AI policy and regulatory
	E. 1. Leadership engagement	Leadership	Management and engagement
	E. 2. Available resources	Data availability	Data quality and management
		Available resources	Finance and resources
	E. 3. Access to knowledge and information	Experiences and prior knowledge	Background
		Education of workforce	Management and engagement
		Interpretability	Trust and transparency
		Documentation and presentation of results	Technical design
IV. CHARACTERISTICS OF INDIVIDUALS	A. Knowledge and belief about the innovation	Experiences and prior knowledge	Background
		Interpretability	Trust and transparency
		Trust	Trust and transparency
	E. Other personal attributes	Motivation	Management and engagement
V. PROCESS	A. Planning	Planning	Management and engagement
	B. 3. Champions	Champions	Management and engagement
	B. 5. Key stakeholders	Involvement	Management and engagement
		Motivation	Management and engagement
	D. Reflecting and evaluating	Evaluation and testing	Evaluation and testing
		Feedback incorporation	Management and engagement
		Model Interoperability	Interoperability
