# Supplementary material for: Artificial Intelligence Implementation in Healthcare: A Theory-Based Scoping Review of Barriers and Facilitators
Source: Int J Environ Res Public Health. 2022 Dec 6;19(23):16359. doi: 10.3390/ijerph192316359 (PMC9738234; doi:10.3390/ijerph192316359)
Supplement: Supplementary file 1 [file ijerph-19-16359-s001.zip › Additional file S5.pdf]

CFIR Codebook

Note: This is a codebook from CFIR Wiki annotated with some examples for our case: AI implementation in healthcare.

**I. Innovation Characteristics**	
A. Innovation Source	Definition: Perception of key stakeholders about whether the innovation is externally or internally developed.<br><br>Inclusion Criteria: Include statements about the source of the innovation and the extent to which interviewees view the change as internal to the organization, e.g., an internally developed program, or external to the organization, e.g., a program coming from the outside. Note: May code and rate as "I" for internal or "E" for external.<br><br>Exclusion Criteria: Exclude or double code statements related to who participated in the decision process to implement the innovation to Engaging, as an indication of early (or late) engagement. Participation in decision-making is an effective engagement strategy to help people feel ownership of the innovation.
B. Evidence Strength & Quality	Definition: Stakeholders' perceptions of the quality and validity of evidence supporting the belief that the innovation will have desired outcomes.<br><br>Inclusion Criteria: Include statements regarding awareness of evidence and the strength and quality of evidence, as well as the absence of evidence or a desire for different types of evidence, such as pilot results instead of evidence from the literature.<br><br>Exclusion Criteria: Exclude or double code statements regarding the receipt of evidence as an engagement strategy to Engaging: Key Stakeholders.<br><br>Exclude or double code descriptions of use of results from local or regional pilots to Trialability.

	Case examples: Here we included statements regarding adjustments made to the product resulting in a trade-off between interpretability and positive predictive (PPV).<br><br>Some papers mentioned published successful work, performance scores better than conventional methods, observable outcomes such as reduced mortality and readmission, generic measures such as QUALYs (Quality-adjusted life-year), and real healthcare setting as evidence facilitating implementation (1, 2)
C. Relative Advantage	Definition: Stakeholders' perception of the advantage of implementing the innovation versus an alternative solution.<br><br>Inclusion Criteria: Include statements that demonstrate the innovation is better (or worse) than existing programs.<br><br>Exclusion Criteria: Exclude statements that demonstrate a strong need for the innovation and/or that the current situation is untenable and code to Tension for Change.
D. Adaptability	Definition: The degree to which an innovation can be adapted, tailored, refined, or reinvented to meet local needs.<br><br>Inclusion Criteria: Include statements regarding the (in)ability to adapt the innovation to their context, e.g., complaints about the rigidity of the protocol. Suggestions for improvement can be captured in this code but should not be included in the rating process, unless it is clear that the participant feels the change is needed but that the program cannot be adapted. However, it may be possible to infer that a large number of suggestions for improvement demonstrates lack of compatibility, see exclusion criteria below.<br><br>Exclusion Criteria: Exclude or double code statements that the innovation did or did not need to be adapted to Compatibility.

	Case examples: Statements regarding the need to adjust for local data and workflow uncovered a need for an additional sub-category called generalizability. If a product needs big adjustments, it can't always be easily transferred across devices and locations.<br>The possibility for site-specific adaptability, customization and predictive capabilities facilitates implementation.<br>Continuously updating and improving the software and systems based on feedback from leaders, users, and the system itself was mentioned in several studies as important (3-5).<br>Adaptability of treatment: Not many AI's deliver treatment, but one study described a product where conversations written by specialist for a chatbot to deliver were personalized for each employee's need. The service platform was also customizable to facilitate care based on unique concerns, cultural values, location and other demographic factors (6).
E. Trialability	Definition: The ability to test the innovation on a small scale in the organization, and to be able to reverse course (undo implementation) if warranted.<br><br>Inclusion Criteria: Include statements related to whether the site piloted the innovation in the past or has plans to in the future, and comments about whether they believe it is (im)possible to conduct a pilot.<br><br>Exclusion Criteria: Exclude or double code descriptions of use of results from local or regional pilots to Evidence Strength & Quality.<br><br>Case examples: One study described a ramp-up period following implementation to test and refine interface and workflow as beneficial (7). Here, a Quality Improvement Team incorporated feedback from leadership and users. Joerin et al. (6) tested their system on a subset and evaluated before incorporating into the treatment.

F. Complexity	Definition: Perceived difficulty of the innovation, reflected by duration, scope, radicalness, disruptiveness, centrality, and intricacy and number of steps required to implement.<br><br>Inclusion Criteria: Code statements regarding the complexity of the innovation itself.<br><br>Exclusion Criteria: Exclude statements regarding the complexity of implementation and code to the appropriate CFIR code, e.g., difficulties related to space are coded to Available Resources and difficulties related to engaging participants in a new program are coded to Engaging: Innovation Participants.<br><br>Case examples: An easy-to use system is easier to implement. A focused, technically modest goal was also mentioned in one study where implementation has been successful. A limited number of variables measured eases application in different hospital settings and enables testing of separate variables fast (1, 5, 8).<br>One study involved a care robot, and here a technologically competent nurse with an understanding of the role as a facilitator of connection was underlined as crucial to successful implementation (2).
G. Design Quality & Packaging	Definition: Perceived excellence in how the innovation is bundled, presented, and assembled.<br><br>Inclusion Criteria: Include statements regarding the quality of the materials and packaging.<br><br>Exclusion Criteria: Exclude statements regarding the presence or absence of materials and code to Available Resources.<br><br>Exclude statements regarding the receipt of materials as an engagement strategy and code to Engaging.<br><br>Case examples: To better suit the nature of implementations studied, quality and structure of user

	interfaces in the product are included here.
H. Cost	<u>Definition</u>: Costs of the innovation and costs associated with implementing the innovation including investment, supply, and opportunity costs.<br><br><u>Inclusion Criteria</u>: Include statements related to the cost of the innovation and its implementation.<br><br><u>Exclusion Criteria</u>: Exclude statements related to physical space and time, and code to <u>Available Resources</u>. In a research study, exclude statements related to costs of conducting the research components (e.g., funding for research staff, participant incentives).
**II. Outer Setting**	
A. Needs & Resources of Those Served by the Organization	<u>Definition</u>: The extent to which the needs of those served by the organization (e.g., patients), as well as barriers and facilitators to meet those needs, are accurately known and prioritized by the organization.<br><br><u>Inclusion Criteria</u>: Include statements demonstrating (lack of) awareness of the needs and resources of those served by the organization. Analysts may be able to infer the level of awareness based on statements about: 1. Perceived need for the innovation based on the needs of those served by the organization and if the innovation will meet those needs; 2. Barriers and facilitators of those served by the organization to participating in the innovation; 3. Participant feedback on the innovation, i.e., satisfaction and success in a program. In addition, include statements that capture whether or not awareness of the needs and resources of those served by the organization influenced the implementation or adaptation of the innovation.<br><br><u>Exclusion Criteria</u>: Exclude statements that demonstrate a strong need for the innovation and/or that the current situation is untenable and code to <u>Tension for Change</u>.<br><br>Exclude statements related to engagement strategies and outcomes, e.g., how innovation participants became

	engaged with the innovation, and code to Engaging: Innovation Participants.<br><br>Case examples: Some studies can report prior work to understand the needs of their local community. After testing content on a subpopulation, evaluation lessons where incorporated. The user experience was extensively and systematically tested to optimize the conversation flow and error management (4).
B. Cosmopolitanism	Definition: The degree to which an organization is networked with other external organizations.<br><br>Inclusion Criteria: Include descriptions of outside group memberships and networking done outside the organization.<br><br>Exclusion Criteria: Exclude statements about general networking, communication, and relationships in the organization, such as descriptions of meetings, email groups, or other methods of keeping people connected and informed, and statements related to team formation, quality, and functioning, and code to Networks & Communications.<br><br>Case examples: Some hospitals will have a network of partners or group of healthcare centers. One study mentioned they had partnered with other groups for testing (4).
C. Peer Pressure	Definition: Mimetic or competitive pressure to implement an innovation, typically because most or other key peer or competing organizations have already implemented or are in a bid for a competitive edge.<br><br>Inclusion Criteria: Include statements about perceived pressure or motivation from other entities or organizations in the local geographic area or system to implement the innovation.<br><br>Exclusion Criteria:

D. External Policy & Incentives	Definition: A broad construct that includes external strategies to spread innovations including policy and regulations (governmental or other central entity), external mandates, recommendations and guidelines, pay-for-performance, collaboratives, and public or benchmark reporting.<br><br>Inclusion Criteria: Include descriptions of external performance measures from the system.<br><br>Exclusion Criteria:<br><br>Case examples: The consideration of AI software as a medical device and the requirement to get the CE mark in accordance to EU/national rules increases cost. The strategy of many hospitals is to present the AI system as a testing of the software, to avoid the need for certification. This hampers the reuse and dissemination of the models built for CDSS (9).
**III. Inner Setting**	
A. Structural Characteristics	Definition: The social architecture, age, maturity, and size of an organization.<br><br>Inclusion Criteria:<br><br>Exclusion Criteria:
B. Networks & Communications	Definition: The nature and quality of webs of social networks, and the nature and quality of formal and informal communications within an organization.<br><br>Inclusion Criteria: Include statements about general networking, communication, and relationships in the organization, such as descriptions of meetings, email groups, or other methods of keeping people connected and informed, and statements related to team formation, quality, and functioning.<br><br>Exclusion Criteria: Exclude statements related to implementation leaders' and users' access to knowledge

	and information regarding using the program, i.e., training on the mechanics of the program and code to Access to Knowledge & Information.<br><br>Exclude statements related to engagement strategies and outcomes, e.g., how key stakeholders became engaged with the innovation and what their role is in implementation, and code to Engaging: Key Stakeholders.<br><br>Exclude descriptions of outside group memberships and networking done outside the organization and code to Cosmopolitanism.<br><br>Case examples: Multidisciplinary teams are an important facilitator for implementation. Assembly and communication of a multidisciplinary team of clinical, research, IT, informatics experts (9).
C. Culture	Definition: Norms, values, and basic assumptions of a given organization.<br><br>Inclusion Criteria: Inclusion criteria, and potential sub-codes, will depend on the framework or definition used for "culture." For example, if using the Competing Values Framework (CVF), you may include four sub-codes related to the four dimensions of the CVF and code statements regarding one or more of the four dimension in an organization.<br><br>Exclusion Criteria:
D. Implementation Climate	Definition: The absorptive capacity for change, shared receptivity of involved individuals to an innovation, and the extent to which use of that innovation will be rewarded, supported, and expected within their organization.<br><br>Inclusion Criteria: Include statements regarding the general level of receptivity to implementing the innovation.<br><br>Exclusion Criteria: Exclude statements regarding the

	general level of receptivity that are captured in the sub-codes.
1. Tension for Change	Definition: The degree to which stakeholders perceive the current situation as intolerable or needing change.<br><br>Inclusion Criteria: Include statements that (do not) demonstrate a strong need for the innovation and/or that the current situation is untenable, e.g., statements that the innovation is absolutely necessary or that the innovation is redundant with other programs. Note: If a participant states that the innovation is redundant with a preferred existing program, (double) code lack of Relative Advantage, see exclusion criteria below.<br><br>Exclusion Criteria: Exclude statements regarding specific needs of individuals that demonstrate a need for the innovation, but do not necessarily represent a strong need or an untenable status quo, and code to Needs and Resources of Those Served by the Organization.<br><br>Exclude statements that demonstrate the innovation is better (or worse) than existing programs and code to Relative Advantage.
2. Compatibility	Definition: The degree of tangible fit between meaning and values attached to the innovation by involved individuals, how those align with individuals' own norms, values, and perceived risks and needs, and how the innovation fits with existing workflows and systems.<br><br>Inclusion Criteria: Include statements that demonstrate the level of compatibility the innovation has with organizational values and work processes. Include statements that the innovation did or did not need to be adapted as evidence of compatibility or lack of compatibility.<br><br>Exclusion Criteria: Exclude or double code statements regarding the priority of the innovation based on compatibility with organizational values to Relative

	Priority, e.g., if an innovation is not prioritized because it is not compatible with organizational values.<br><br>Case examples: ML is feared as it may introduce important biases, proper evaluation (including qualitative by clinicians) should be planned. AI systems can be thus be viewed as introducing a real risk into clinical practice (5). In addition the lack of integration with the EHR data can be an issue (5).
3. Relative Priority	Definition: Individuals' shared perception of the importance of the implementation within the organization.<br><br>Inclusion Criteria: Include statements that reflect the relative priority of the innovation, e.g., statements related to change fatigue in the organization due to implementation of many other programs.<br><br>Exclusion Criteria: Exclude or double code statements regarding the priority of the innovation based on compatibility with organizational values to Compatibility, e.g., if an innovation is not prioritized because it is not compatible with organizational values.
4. Organizational Incentives & Rewards	Definition: Extrinsic incentives such as goal-sharing, awards, performance reviews, promotions, and raises in salary, and less tangible incentives such as increased stature or respect.<br><br>Inclusion Criteria: Include statements related to whether organizational incentive systems are in place to foster (or hinder) implementation, e.g., rewards or disincentives for staff engaging in the innovation.<br><br>Exclusion Criteria:
5. Goals & Feedback	Definition: The degree to which goals are clearly communicated, acted upon, and fed back to staff, and alignment of that feedback with goals.<br><br>Inclusion Criteria: Include statements related to the (lack of) alignment of implementation and innovation goals

	with larger organizational goals, as well as feedback to staff regarding those goals, e.g., regular audit and feedback showing any gaps between the current organizational status and the goal. Goals and Feedback include organizational processes and supporting structures independent of the implementation process. Evidence of the integration of evaluation components used as part of "Reflecting and Evaluating" into **on-going or sustained** organizational structures and processes may be (double) coded to Goals and Feedback.<br><br>Exclusion Criteria: Exclude statements that refer to the implementation team's (lack of) assessment of the progress toward and impact of implementation, as well as the interpretation of outcomes related to implementation, and code to Reflecting & Evaluating. Reflecting and Evaluating is part of the implementation process; it likely ends when implementation activities end. It does not require goals be explicitly articulated; it can focus on descriptions of the current state with real-time judgment, though there may be an implied goal (e.g., we need to implement the innovation) when the implementation team discusses feedback in terms of adjustments needed to complete implementation.
6. Learning Climate	Definition: A climate in which: 1. Leaders express their own fallibility and need for team members' assistance and input; 2. Team members feel that they are essential, valued, and knowledgeable partners in the change process; 3. Individuals feel psychologically safe to try new methods; and 4. There is sufficient time and space for reflective thinking and evaluation.<br><br>Inclusion Criteria: Include statements that support (or refute) the degree to which key components of an organization exhibit a "learning climate."<br><br>Exclusion Criteria:
E. Readiness for Implementation	Definition: Tangible and immediate indicators of organizational commitment to its decision to implement an

	innovation.<br><br><u>Inclusion Criteria</u>: Include statements regarding the general level of readiness for implementation.<br><br><u>Exclusion Criteria</u>: Exclude statements regarding the general level of readiness for implementation that are captured in the sub-codes.<br><br><u>Case examples:</u> Training of nurses and other staff members before implementation (3, 5)
1. Leadership Engagement	<u>Definition</u>: Commitment, involvement, and accountability of leaders and managers with the implementation of the innovation.<br><br><u>Inclusion Criteria</u>: Include statements regarding the level of engagement of organizational leadership.<br><br><u>Exclusion Criteria</u>: Exclude or double code statements regarding leadership engagement to Engaging: <u>Formally Appointed Internal Implementation Leaders</u> or <u>Champions</u> *if* an organizational leader is also an implementation leader, e.g., if a director of primary care takes the lead in implementing a new treatment guideline. Note that a key characteristic of this Implementation Leader/Champion is that s/he is also an Organizational Leader.
2. Available Resources	<u>Definition</u>: The level of resources organizational dedicated for implementation and on-going operations including physical space and time.<br><br><u>Inclusion Criteria</u>: Include statements related to the presence or absence of resources specific to the innovation that is being implemented.<br><br><u>Exclusion Criteria</u>: Exclude statements related to training and education and code to <u>Access to Knowledge & Information</u>.<br><br>Exclude statements related to the quality of materials and

	code to Design Quality & Packaging.<br><br>In a research study, exclude statements related to resources needed for conducting the research components (e.g., time to complete research tasks, such as IRB applications, consenting patients).<br><br>Case examples: One of the largest resource required for the successful translation of AI into clinical routine is personnel time, for example in this sepsis watch implementation (4).<br><br>"Key data that reliably predict the outcome of interest may not be readily available as structured, discrete data inputs from the EHR" (7)
3. Access to Knowledge & Information	Definition: Ease of access to digestible information and knowledge about the innovation and how to incorporate it into work tasks.<br><br>Inclusion Criteria: Include statements related to implementation leaders' and users' access to knowledge and information regarding use of the program, i.e., training on the mechanics of the program.<br><br>Exclusion Criteria: Exclude statements related to engagement strategies and outcomes, e.g., how key stakeholders became engaged with the innovation and what their role is in implementation, and code to Engaging: Key Stakeholders.<br><br>Exclude statements about general networking, communication, and relationships in the organization, such as descriptions of meetings, email groups, or other methods of keeping people connected and informed, and statements related to team formation, quality, and functioning, and code to Networks & Communications.<br><br>Case examples: Lack of knowledge of the clinical workflow

	can hamper readiness for implementation (5).
**IV. Characteristics of Individuals**	
1. Knowledge & Beliefs about the Innovation	Definition: Individuals' attitudes toward and value placed on the innovation, as well as familiarity with facts, truths, and principles related to the innovation.<br><br>Inclusion Criteria:<br><br>Exclusion Criteria: Exclude statements related to familiarity with evidence about the innovation and code to Evidence Strength & Quality.<br><br>Case examples: This is a critical factor, because some beliefs about AI may have been altered by difficult early AI systems, therefore physicians can be skeptical (2).
2. Self-efficacy	Definition: Individual belief in their own capabilities to execute courses of action to achieve implementation goals.<br><br>Inclusion Criteria:<br><br>Exclusion Criteria:
3. Individual Stage of Change	Definition: Characterization of the phase an individual is in, as s/he progresses toward skilled, enthusiastic, and sustained use of the innovation.<br><br>Inclusion Criteria:<br><br>Exclusion Criteria:
4. Individual Identification with Organization	Definition: A broad construct related to how individuals perceive the organization, and their relationship and degree of commitment with that organization.<br><br>Inclusion Criteria:<br><br>Exclusion Criteria:
5. Other Personal Attributes	Definition: A broad construct to include other personal traits such as tolerance of ambiguity, intellectual ability, motivation, values, competence, capacity, and learning

	style.<br><br>Inclusion Criteria:<br><br>Exclusion Criteria:<br>Case examples: Nurses as intermediate between robot and patient requires technology competency and an understanding of their role as a facilitator of connection (2).
**V. Process**	
A. Planning	Definition: The degree to which a scheme or method of behavior and tasks for implementing an innovation are developed in advance, and the quality of those schemes or methods.<br><br>Inclusion Criteria: Include evidence of pre-implementation diagnostic assessments and planning, as well as refinements to the plan.<br><br>Exclusion Criteria:<br><br>Case examples: Involvement of clinical stuff in design and development and clinical governance committee from the inception of the project. Organization of roles, promotion, training, communication, workflow definition, evaluation and post-implementation sustainability planning (2). Prior to the trial, the project team collaborated with local practice partners to map local workflows and identify how to best to implement the tool (7). In the planning, the implementation was divided into 3 phases; (i) preliminary launch, (ii) user experience optimization and service scaling, (iii) extend support to patients.
B. Engaging	Definition: Attracting and involving appropriate individuals in the implementation and use of the innovation through a combined strategy of social marketing, education, role modeling, training, and other similar activities.

	Inclusion Criteria: Include statements related to engagement strategies and outcomes, i.e., if and how staff and innovation participants became engaged with the innovation and what their role is in implementation. Note: Although both strategies and outcomes are coded here, the outcome of engagement efforts determines the rating, i.e., if there are repeated attempts to engage staff that are unsuccessful, or if a role is vacant, the construct receives a negative rating. In addition, you may also want to code the "quality" of staff - their capabilities, motivation, and skills, i.e., how good they are at their job, and this data affects the rating as well.<br><br>Exclusion Criteria: Exclude statements related to specific sub constructs, e.g., Champions or Opinion Leaders.<br><br>Exclude or double code statements related to who participated in the decision process to implement the innovation to Innovation Source, as an indicator of internal or external innovation source.
1. Opinion Leaders	Definition: Individuals in an organization that have formal or informal influence on the attitudes and beliefs of their colleagues with respect to implementing the innovation.<br><br>Inclusion Criteria: Include statements related to engagement strategies and outcomes, e.g., how the opinion leader became engaged with the innovation and what their role is in implementation. Note: Although both strategies and outcomes are coded here, the outcome of efforts to engage staff determines the rating, i.e., if there are repeated attempts to engage an opinion leader that are unsuccessful, or if the opinion leader leaves the organization and this role is vacant, the construct receives a negative rating. In addition, you may also want to code the "quality" of the opinion leader here - their capabilities, motivation, and skills, i.e., how good they are at their job, and this data affects the rating as well.<br><br>Exclusion Criteria:

2. Formally Appointed Internal Implementation Leaders	Definition: Individuals from within the organization who have been formally appointed with responsibility for implementing an innovation as coordinator, project manager, team leader, or other similar role.<br><br>Inclusion Criteria: Include statements related to engagement strategies and outcomes, e.g., how the formally appointed internal implementation leader became engaged with the innovation and what their role is in implementation. Note: Although both strategies and outcomes are coded here, the outcome of efforts to engage staff determines the rating, i.e., if there are repeated attempts to engage an implementation leader that are unsuccessful, or if the implementation leader leaves the organization and this role is vacant, the construct receives a negative rating. In addition, you may also want to code the "quality" of the implementation leader here - their capabilities, motivation, and skills, i.e., how good they are at their job, and this data affects the rating as well.<br><br>Exclusion Criteria: Exclude or double code statements regarding leadership engagement to Leadership Engagement *if* an implementation leader is also an organizational leader, e.g., if a director of primary care takes the lead in implementing a new treatment guideline.
3. Champions	Definition: "Individuals who dedicate themselves to supporting, marketing, and 'driving through' an [implementation]", overcoming indifference or resistance that the innovation may provoke in an organization.<br><br>Inclusion Criteria: Include statements related to engagement strategies and outcomes, e.g., how the champion became engaged with the innovation and what their role is in implementation. Note: Although both strategies and outcomes are coded here, the outcome of efforts to engage staff determines the rating, i.e., if there are repeated attempts to engage a champion that are unsuccessful, or if the champion leaves the organization and this role is vacant, the construct receives a negative

	rating. In addition, you may also want to code the "quality" of the champion here - their capabilities, motivation, and skills, i.e., how good they are at their job, and this data affects the rating as well.<br><br>Exclusion Criteria: Exclude or double code statements regarding leadership engagement to Leadership Engagement *if* a champion is also an organizational leader, e.g., if a director of primary care takes the lead in implementing a new treatment guideline.<br><br>Case examples: There can be assigned a team, such as the clinical governance committee (4), that can be clinical champions who promote the use of the system.
4. External Change Agents	Definition: Individuals who are affiliated with an outside entity who formally influence or facilitate innovation decisions in a desirable direction.<br><br>Inclusion Criteria: Include statements related to engagement strategies and outcomes, e.g., how the external change agent (entities outside the organization that facilitate change) became engaged with the innovation and what their role is in implementation, e.g., how they supported implementation efforts. Note: Although both strategies and outcomes are coded here, the outcome of efforts to engage staff determines the rating, i.e., if there are repeated attempts to engage an external change agent that are unsuccessful, or if the external change agent leaves their organization and this role is vacant, the construct receives a negative rating. In addition, you may also want to code the "quality" of the external change agent here - their capabilities, motivation, and skills, i.e., how good they are at their job, and this data affects the rating as well.<br><br>Exclusion Criteria: Note: It is important to clearly define what roles are external and internal to the organization. Exclude statements regarding facilitating activities, such as training in the mechanics of the program, and code to Access to Knowledge & Information *if* the change agent is considered internal to the study, e.g., a staff member at the

	national office. If the study considers this staff member internal to the organization, it should be coded to Access to Knowledge & Information, even though their support may overlap with what would be expected from an External Change Agent.
5. Key Stakeholders	Definition: Individuals from within the organization that are directly impacted by the innovation, e.g., staff responsible for making referrals to a new program or using a new work process.<br><br>Inclusion Criteria: Include statements related to engagement strategies and outcomes, e.g., how key stakeholders became engaged with the innovation and what their role is in implementation. Note: Although both strategies and outcomes are coded here, the outcome of efforts to engage staff determines the rating, i.e., if there are repeated attempts to engage key stakeholders that are unsuccessful, the construct receives a negative rating.<br><br>Exclusion Criteria: Exclude statements related to implementation leaders' and users' access to knowledge and information regarding using the program, i.e., training on the mechanics of the program, and code to Access to Knowledge & Information.<br><br>Exclude statements about general networking, communication, and relationships in the organization, such as descriptions of meetings, email groups, or other methods of keeping people connected and informed, and statements related to team formation, quality, and functioning, and code to Networks & Communications.
6. Innovation Participants	Definition: Individuals served by the organization that participate in the innovation, e.g., patients in a prevention program in a hospital.<br><br>Inclusion Criteria: Include statements related to engagement strategies and outcomes, e.g., how innovation participants became engaged with the innovation. Note: Although both strategies and outcomes are coded here, the

	outcome of efforts to engage participants determines the rating, i.e., if there are repeated attempts to engage participants that are unsuccessful, the construct receives a negative rating.<br><br>Exclusion Criteria: Exclude statements demonstrating (lack of) awareness of the needs and resources of those served by the organization and whether or not that awareness influenced the implementation or adaptation of the innovation and code to Needs & Resources of Those Served by the Organization.
C. Executing	Definition: Carrying out or accomplishing the implementation according to plan.<br><br>Inclusion Criteria: Include statements that demonstrate how implementation occurred with respect to the implementation plan. Note: Executing is coded very infrequently due to a lack of planning. However, some studies have used fidelity measures to assess executing, as an indication of the degree to which implementation was accomplished according to plan.<br><br>Exclusion Criteria:
D. Reflecting & Evaluating	Definition: Quantitative and qualitative feedback about the progress and quality of implementation accompanied with regular personal and team debriefing about progress and experience.<br><br>Inclusion Criteria: Include statements that refer to the implementation team's (lack of) assessment of the progress toward and impact of implementation, as well as the interpretation of outcomes related to implementation. Reflecting and Evaluating is part of the implementation process; it likely ends when implementation activities end. It does not require goals be explicitly articulated; it can focus on descriptions of the current state with real-time judgment, though there may be an implied goal (e.g., we need to implement the innovation) when the implementation team discusses feedback in terms of adjustments needed to complete implementation.

	Exclusion Criteria: Exclude statements related to the (lack of) alignment of implementation and innovation goals with larger organizational goals, as well as feedback to staff regarding those goals, e.g., regular audit and feedback showing any gaps between the current organizational status and the goal, and code to Goals & Feedback. Goals and Feedback include organizational processes and supporting structures independent of the implementation process. Evidence of the integration of evaluation components used as part of "Reflecting and Evaluating" into **on-going or sustained** organizational structures and processes may be (double) coded to Goals and Feedback.<br><br>Exclude statements that capture reflecting and evaluating that participants may do during the interview, for example, related to the success of the implementation, and code to Knowledge & Beliefs about the Innovation.<br><br>Case examples: Some organization can assemble a post-implementation team to hold regular scheduled feedback meetings to discuss systemic improvements, for example, the primary area for improvement in one study was concerned with the algorithm threshold (3). Some organizations can conduct post-implementation clinical trials to confirm the impact of the new system (10).
**VI. Additional Codes**	
A. Code Name	Definition:<br><br>Inclusion Criteria:<br><br>Exclusion Criteria:
B. Code Name	Definition:<br><br>Inclusion Criteria:<br><br>Exclusion Criteria:

General Coding Rules:

When two codes are in question for a passage, consider the primary meaning of the passage to assign code; consider what the participant is truly saying. Analysts may wish to err on the side of inclusion or double coding.

General Rating Rules:

Ratings						
M	-2	-1	0	X	+1	+2

In general, ratings are determined based on two factors: 1) valence and 2) strength.

Valence: positive or negative influence on implementation
Rating component: X, 0, +, -
The valence component of a rating is determined by the influence the coded data has on the implementation process, i.e., contextual factors that facilitate or hinder implementation. Due to limited data, analysts may have to infer the influence on implementation based on simple presence or absence of a construct. For example, if a participant states that the intervention has advantages over existing programs, but does not state how this has influenced implementation, the analyst can infer that the presence of relative advantage facilitated implementation. However, whenever the data allows, the analysts should apply ratings based on the influence the construct has on implementation, not the presence or absence of a construct; presence or absence of a positive construct (e.g. relative advantage) does not always constitute a matching positive or negative influence on implementation.

In the event that comments are mixed, i.e., some comments are negative and some comments are positive, try to tip the rating to a weak positive or weak negative, based on the aggregate of the comments. However, if you feel the comments are equally positive and negative, apply a mixed (X) rating. Some users of the CFIR have denoted level of agreement among participants in their rating by adding a * to the rating if comments were mixed. For example, if the aggregate of mixed comments was positive, the rating was +1*. Some users feel it's important to record discord among participants because it indicates a negative influence on implementation.

In the event that the comments are neutral, i.e., comments are related to a construct but have no bearing on the implementation, apply the neutral (0) rating.

Strength: weak or strong influence on implementation
Rating component: 1, 2

The strength component of a rating is determined by a number of factors, including: level of agreement among participants, strength of language, and use of concrete examples. However, sometimes analysts may choose to apply relative ratings, versus absolute ratings, in order to differentiate between organization in the study.

1. Scheinker D, Brandeau ML. Implementing analytics projects in a hospital: successes, failures, and opportunities. INFORMS Journal on Applied Analytics. 2020;50(3):176-89.
2. Betriana F, Tanioka T, Osaka K, Kawai C, Yasuhara Y, Locsin RC. Interactions between healthcare robots and older people in Japan: A qualitative descriptive analysis study. Japan Journal of Nursing Science. 2021:e12409.
3. McCoy A, Das R. Reducing patient mortality, length of stay and readmissions through machine learning-based sepsis prediction in the emergency department, intensive care unit and hospital floor units. BMJ open quality. 2017;6(2):e000158.
4. Sendak MP, Ratliff W, Sarro D, Alderton E, Futoma J, Gao M, et al. Real-world integration of a sepsis deep learning technology into routine clinical care: implementation study. JMIR medical informatics. 2020;8(7):e15182.
5. Xu H, Li P, Yang Z, Liu X, Wang Z, Yan W, et al. Construction and application of a medical-grade wireless monitoring system for physiological signals at general wards. Journal of Medical Systems. 2020;44(10):1-15.
6. Joerin A, Rauws M, Ackerman ML. Psychological artificial intelligence service, Tess: delivering on-demand support to patients and their caregivers: technical report. Cureus. 2019;11(1).
7. Romero-Brufau S, Wyatt KD, Boyum P, Mickelson M, Moore M, Cognetta-Rieke C. Implementation of artificial intelligence-based clinical decision support to reduce hospital readmissions at a regional hospital. Applied Clinical Informatics. 2020;11(04):570-7.
8. Cho K-J, Kwon O, Kwon J-m, Lee Y, Park H, Jeon K-H, et al. Detecting patient deterioration using artificial intelligence in a rapid response system. Critical care medicine. 2020;48(4):e285-e9.
9. Schuh C, de Bruin JS, Seeling W. Clinical decision support systems at the Vienna General Hospital using Arden Syntax: Design, implementation, and integration. Artif Intell Med. 2018;92:24-33.
10. Petitgand C, Motulsky A, Denis J-L, Régis C. Investigating the barriers to physician adoption of an artificial intelligence-based decision support system in emergency care: an interpretative qualitative study. Digital Personalized Health and Medicine: IOS Press; 2020. p. 1001-5.
